# Mastisol

**Published:** 1915-11

**Authors:** 


**Mastisol.** The Prescriber gives the following formulae for this varnish dressing largely used in the German army. The second formula is said to be better: Mastic 40., G. Benzene 60., G. Castor oil 20 drops. Mastic 20, Resin 10, Turpentine (oleo-resin) 7, Benzene 50.

